# Supplementary material for: Speaking up behavior and cognitive bias in hand hygiene: Competences of German-speaking medical students
Source: PLoS One. 2020 Sep 28;15(9):e0239444. doi: 10.1371/journal.pone.0239444 (PMC7521694; doi:10.1371/journal.pone.0239444)
Supplement: S1 File — (PDF) [file pone.0239444.s002.pdf]

Medizinische Ausbildung in der Infektionsprävention

Umfrage zu Infektionsprävention und Patientensicherheit

Sehr geehrte Studierende,

hiermit möchten wir Sie zu einer etwa 8 bis 12 minütigen anonymen Umfrage zur Ausbildung in der Infektionsprävention einladen und danken Ihnen bereits jetzt für Ihre Teilnahme.

Hintergrund unseres Vorhabens ist die Prävention von Infektionen,die im Krankenhaus erworben werden. Besonders die Infektionen mit multiresistenten Erregern (MRE) wie MRSA, VRE und MRGN stellen uns und Sie als zukünftige Kolleginnen und Kollegen in den nächsten Jahren vor große Herausforderungen, sodass eine ständige Anpassung der Aus- und späteren Weiter- und Fortbildung notwendig ist.

Mit dieser Umfrage möchten wir Ihre Einschätzungen zu den eigenen Kompetenzen im Bereich von Infektionspräventionen mit der Einschätzung anderer Personen ermitteln: Hierzu zählen zum einen manuelle Fertigkeiten wie die Händehygiene, aber auch das korrigierende Eingreifen bei beobachteten Fehlern.

Mit dieser anonymen (keine Speicherung von Name oder IP-Adresse) Umfrage des Instituts für Krankenhaushygiene und Infektionsprävention (IKIP) des Gesundheitsverbundes Landkreis Konstanz (GLKN) und dem Universitätsklinikum Regensburg wollen wir diese Einschätzungen erfassen und zur wissenschaftlichen Auswertung nutzen.

Unser Team handelt unabhängig von Fachgesellschaften, Marktforschung und der Industrie (kein Conflict of Interest).

Ihre Antworten werden vertraulich behandelt. Die Gesamtergebnisse sind zur Publikation in einer medizinischen Fachzeitschrift vorgesehen.

Bei Fragen zur Umfrage oder zum Thema wenden Sie sich bitte an den Studienleiter (stefan.bushuven [a] glkn.de)

Wir danken Ihnen für die Teilnahme!

Mit kollegialen Grüßen
Stefan Bushuven

Dr.med. Stefan Bushuven
Facharzt für Anästhesiologie, ZB Medizinhygiene, Intensiv- und Notfallmedizin
Krankenhaushygieniker am Institut für Krankenhaushygiene und Infektionsprävention
Gesundheitsverbund Landkreis Konstanz

Email: stefan[punkt[bushuven[ad]glkn.de

Zuerst einige Fragen an Sie ...

In welchem Studiensemester befinden Sie sich gerade (1.-12.) ? *

(wenn Sie Semester wiederholt haben, geben Sie bitte das Semester an, nach dessen Stundenplan Sie aktuell Ihr Studium ausrichten)

Welches Geschlecht haben Sie? *

- weiblich
- männlich
- divers/transgender/transidentical
- keine Angabe

Wie alt sind Sie? *

möchten Sie Ihr Alter nicht angeben, tragen Sie bitte "1" ein

Wo studieren Sie aktuell?

freiwillige Angabe

Haben Sie den Studienort schon einmal gewechselt ? *

◯ ja

◯ nein

Haben Sie bereits eine abgeschlossene Ausbildung in einem medzinischen Beruf? *

z.B. Rettungsdienst (RA, NotSan, aber nicht RS), Gesundheits- und Krankenpflege, OTA, ATA, CTA, MTA, MFA, Physio-/Ergo-Therapie, Logopädie, medizinische andere Studienfächer

◯ ja

◯ nein

Welchen Studiengang absolvieren Sie derzeit? *

◯ Humanmedizin

◯ Zahnmedizin

◯ keine Angabe

In welchem Semester erfolgte Ihre erste Unterweisung in der Händehygiene? *

Bitte wählen... ▾

In welchem Semester erfolgt/erfolgte gemäß Lehrplan die erste Lehrveranstaltung zum Thema Krankenhaushygiene Ihrer Fakultät? *

Bitte wählen... ▾

Fragen zur Selbsteinschätzung

Ich führe die hygienische Händedesinfektion situationsgerecht durch. *

- ◯ Trifft voll zu
- ◯ Trifft ziemlich zu
- ◯ Trifft teilweise zu
- ◯ Trifft wenig zu
- ◯ Trifft nicht zu

Ich erkenne die Indikationen für eine hygienische Händedesinfektion. *

- ◯ Trifft voll zu
- ◯ Trifft ziemlich zu
- ◯ Trifft teilweise zu
- ◯ Trifft wenig zu
- ◯ Trifft nicht zu

Ich wähle das benötigte Händedesinfektionsmittel situationsgerecht aus. *

- ◯ Trifft voll zu
- ◯ Trifft ziemlich zu
- ◯ Trifft teilweise zu
- ◯ Trifft wenig zu
- ◯ Trifft nicht zu

Ich erkenne Fehler in der Durchführung der hygienischen Händedesinfektion bei anderen Personen. *

- ◯ Trifft voll zu
- ◯ Trifft ziemlich zu
- ◯ Trifft teilweise zu
- ◯ Trifft wenig zu
- ◯ Trifft nicht zu

Ich greife korrigierend ein, wenn ich einen Fehler in der Durchführung der hygienischen Händedesinfektion bemerke. *

- ◯ Trifft voll zu
- ◯ Trifft ziemlich zu
- ◯ Trifft teilweise zu
- ◯ Trifft wenig zu
- ◯ Trifft nicht zu

Ich nehme situationsgerecht Rückmeldungen an, wenn ich durch eine andere Person auf einen Fehler in der hygienischen Händedesinfektion hingewiesen werde. *

- ◯ Trifft voll zu
- ◯ Trifft ziemlich zu
- ◯ Trifft teilweise zu
- ◯ Trifft wenig zu
- ◯ Trifft nicht zu

Bitte schätzen Sie das beobachtete Verhalten Ihrer Kommillitonen ein!

Meine Kommillitonen setzen die hygienische Händedesinfektion situationsgerecht um. *

◯ Trifft voll zu ◯ Trifft ziemlich zu ◯ Trifft teilweise zu ◯ Trifft wenig zu ◯ Trifft nicht zu

Meine Kommillitonen wissen die jeweiligen Indikationen für eine hygienische Händedesinfektion *

◯ Trifft voll zu ◯ Trifft ziemlich zu ◯ Trifft teilweise zu ◯ Trifft wenig zu ◯ Trifft nicht zu

Meine Kommillitonen wählen das notwendige Desinfektionsmittel für eine hygienische Händedesinfektion korrekt aus *

◯ Trifft voll zu ◯ Trifft ziemlich zu ◯ Trifft teilweise zu ◯ Trifft wenig zu ◯ Trifft nicht zu

Meine Kommillitonen korrigieren mich situationsgerecht, wenn sie einen Fehler in meiner Händedesinfektion bemerken *

◯ Trifft voll zu ◯ Trifft ziemlich zu ◯ Trifft teilweise zu ◯ Trifft wenig zu ◯ Trifft nicht zu

Meine Kommillitonen nehmen situationsgerecht Rückmeldungen und Hinweise an, wenn sie auf einen Hygienefehler angesprochen werden. *

◯ Trifft voll zu ◯ Trifft ziemlich zu ◯ Trifft teilweise zu ◯ Trifft wenig zu ◯ Trifft nicht zu

Bitte schätzen Sie das beobachtete Verhalten von Ärztinnen und Ärzten ein!

Ärztinnen und Ärzte führen die hygienische Händedesinfektion situationsgerecht aus. *

- ◯ Trifft voll zu
- ◯ Trifft ziemlich zu
- ◯ Trifft teilweise zu
- ◯ Trifft wenig zu
- ◯ Trifft nicht zu

Ärztinnen und Ärzte korrigieren mich situationsgerecht, wenn sie einen Fehler in meiner Händedesinfektion bemerken *

- ◯ Trifft voll zu
- ◯ Trifft ziemlich zu
- ◯ Trifft teilweise zu
- ◯ Trifft wenig zu
- ◯ Trifft nicht zu

Ärztinnen und Ärzte nehmen situationsgerecht Rückmeldungen und Hinweise an, wenn sie auf einen Hygienefehler angesprochen werden. *

- ◯ Trifft voll zu
- ◯ Trifft ziemlich zu
- ◯ Trifft teilweise zu
- ◯ Trifft wenig zu
- ◯ Trifft nicht zu

Bitte schätzen Sie das beobachtete Verhalten von Gesundheits- und Krankenpflegekräften ein!

Pflegekräfte führen die hygienische Händedesinfektion situationsgerecht aus. *

- ◯ Trifft voll zu
- ◯ Trifft ziemlich zu
- ◯ Trifft teilweise zu
- ◯ Trifft wenig zu
- ◯ Trifft nicht zu

Pflegekräfte korrigieren mich situationsgerecht, wenn sie einen Fehler in meiner Händedesinfektion bemerken *

- ◯ Trifft voll zu
- ◯ Trifft ziemlich zu
- ◯ Trifft teilweise zu
- ◯ Trifft wenig zu
- ◯ Trifft nicht zu

Pflegekräfte nehmen situationsgerecht Rückmeldungen und Hinweise an, wenn sie auf einen Hygienefehler angesprochen werden *

- Trifft voll zu
- Trifft ziemlich zu
- Trifft teilweise zu
- Trifft wenig zu
- Trifft nicht zu

Wie schätzen Sie die Gefahr unterlassener Händedesinfektionen ein?

Der glaubwürdig maximale Schaden einer unterlassenen Händedesinfektion ist *

- unbedeutend
- gering - aber keine nachhaltigen Schäden
- spürbar- mit verlängertem Aufenthalt im Krankenhaus
- kritisch - mit bleibenden körperlichen Schäden
- katastrophal - mit Todesfolge
- keine Angabe
- weiß nicht

Wie häufig kommt es in Ihrem Ausbildungs - Umfeld (Universitätsklinikum / Lehrkrankenhaus) vor, dass ein Patient den geschätzten Schaden durch eine ausgebliebene Händedesinfektion erleidet? *

- ◯ seltener als einmal in 3 Jahren
- ◯ häufiger als. einmal in 3 Jahren
- ◯ häufiger als einmal pro Jahr
- ◯ häufiger als einmal pro 3 Monate
- ◯ häufiger als einmal pro Monat
- ◯ keine Angabe
- ◯ weiß ich nicht

Wie schätzen Sie Ihr eigenes Verhalten im medizinischen Alltag ein?

In folgenden Situationen setze ich die hygienische Händedesinfektion um *

Die Prozenanageben beziehen sich auf Ihre Schätzung, wie häufig Sie dies in entsprechenden Fällen in Prozent durchführen

	Immer (>99%)	Häufig (75-99%)	Mittelmäßig (25-75%)	Selten (1-25%)	Niemals (< 1%)	keine Angabe möglich
Nach versehentlicher Kontamination der eigenen Hand mit Urin eines Patienten	○	○	○	○	○	○
Nach dem Aufheben eines heruntergefallenen Handtuchs eines Patienten im Bad	○	○	○	○	○	○
Vor dem Umstecken eines neuen Urin-Beutels an einen Harnblasendauerkatheter	○	○	○	○	○	○
Nach dem Abstellen eines benutzten Patientenbettes in einen Untersuchungsraum	○	○	○	○	○	○
Vor der Begrüßung eines Patienten mit Handschlag	○	○	○	○	○	○
Vor dem Betreten eines Patientenzimmers bei einer Visite	○	○	○	○	○	○
Nach der Verabschiedung eines Patienten mit Handschlag	○	○	○	○	○	○
Vor dem Lagern eines Patienten im Bett oder auf einem OP-Tisch	○	○	○	○	○	○
Vor der Verbindung einer Infusionsleitung an eine periphere Venenverweilkanüle	○	○	○	○	○	○
Nach dem Essen in der Mittagspause	○	○	○	○	○	○
Nach dem Aufhelfen eines gestürzten Patienten	○	○	○	○	○	○
Nach dem Vorbereiten eines Medikaments	○	○	○	○	○	○

Wenn ich einen Fehler in der hygienischen Händedesinfektion bei anderen Personen bemerke, greife ich korrigierend ein bei *

	Trifft voll zu	Trifft ziemlich zu	Trifft teilweise zu	Trifft wenig zu	Trifft nicht zu	Keine Angabe
Besuchern von Patienten	○	○	○	○	○	○
Reinigungskräften	○	○	○	○	○	○
Auszubildenden in der Pflege	○	○	○	○	○	○
Pflegekräften	○	○	○	○	○	○
Kommilitonen	○	○	○	○	○	○
Assistenzärzten	○	○	○	○	○	○
Oberärzten	○	○	○	○	○	○
Chefärzten	○	○	○	○	○	○

Bitte geben Sie Ihre Einschätzung zum Korrekturverhalten ab

Wer greift bei Fehlern anderer Personen ein? *

Bitte Bewerten Sie die Spalten (diese Person wird korrigiert) durch die jeweiligen Korrigierenden (Zeilen). Bewerten Sie auch, ob sich Berufsgruppen gegenseitig korrigieren

BITTE BEWERTEN SIE IN DIESER MATRIX JEDE ZEILE ZU JEDER SPALTE (MEHRFACHAUSWAHL)!

	Besucherinnen und Besucher	Reinigungskräfte	Auszubildende	Gesundheits- und Krankenpflegekräfte	Studierende	Assistenzärztinnen und -ärzte	Oberärztinnen und Oberärzte	Chefärztinnen und Chefärzte	niemanden
Besucher und Besucherinnen korrigieren	☐	☐	☐	☐	☐	☐	☐	☐	☐
Reinigungskräfte korrigieren	☐	☐	☐	☐	☐	☐	☐	☐	☐
Auszubildende in der Pflege korrigieren	☐	☐	☐	☐	☐	☐	☐	☐	☐
Gesundheits- und Krankenpflegekräfte korrigieren	☐	☐	☐	☐	☐	☐	☐	☐	☐
Studierende korrigieren	☐	☐	☐	☐	☐	☐	☐	☐	☐
Assistenzärztinnen und Assistenzärzte korrigieren	☐	☐	☐	☐	☐	☐	☐	☐	☐
Oberärztinnen und Oberärzte korrigieren	☐	☐	☐	☐	☐	☐	☐	☐	☐
Chefärztinnen und Chefärzte korrigieren	☐	☐	☐	☐	☐	☐	☐	☐	☐

Bitte geben Sie Ihre Einschätzung ab

In welchem Semester sollte die Ausbildung in der hygienischen Händedesinfektion beginnen? *

Bitte wählen...

Wie häufig sollte die Ausbildung in der hygienischen Händedesinfektion im Sinne einer "Auffrischung" während des Studiums wiederholt werden? *

Bitte wählen...

Bitte ordnen Sie folgende Ausbildungsinhalte der Patientensicherheit nach ihrer Wichtigkeit! (1= am wichtigsten) *

- Sicherheit in der Anwendung von Medikamenten (Medication Safety)
- Maßnahmen der Infektionsprävention (Krankenhaushygiene und Antibiotic Stewardship)
- Anwendersicherheit in der Diagnostik (Klinisches Bild, Laborparameter, Bildgebung)
- Strategien in der Überwachung (Surveillance) und Therapie der Sepsis (Blutvergiftung)
- Strategien zu Cyber-Sicherheit medizinischer Produkte und Datensschutz
- Strategien zur Verhinderung von unnötigen Krankenhaushaufnahmen

Bitte ordnen Sie die Inhalte in der Krankenhaushygiene und Infektionsprävention nach Wichtigkeit für die ärztliche Ausbildung! (1= am wichtigsten) *

- Umgang mit Persönlicher Schutzausrüstung (Handschuhe, Kittel, Mundschutz, ...)
- Hygienische Händedesinfektion
- Umgang mit Isolierungsmaßnahmen (bei Patienten mit multiresistenten Erregern wie MRSA)
- Flächendesinfektion
- Medizinprodukteaufbereitung (z.B. OP-Instrumente)
- Verhalten im Ausbruchsfalls (Häufung von Infektionserkrankungen)
- Umgang mit der Präanalytik (Wie nehme ich Labor-Proben ab, lagere und versende sie?)
- Diagnostik von Infektionserkrankungen (z.B. klinische Untersuchung, Interpretation von Laborergebnissen, Bildgebung)
- Therapie von Infektionskrankheiten (z.B. Einsatz von Antibiotika)

Verbesserungsvorschläge, Anregungen und Anmerkungen

Haben Sie Verbesserungsvorschläge, spezielle Erfahrungen oder Anmerkungen zu Infektionsprävention und Krankenhaushygiene ? Lassen Sie es uns wissen!

Geschafft! Wollen Sie mehr wissen? Bestehen Fragen oder Unklarheiten?

Sie haben es geschafft!

Vielen Dank!

Wenn Sie Fragen zur hygienischen Händedesinfektion und Infektionsprävention haben, wenden Sie sich an den Studienleiter (stefan[dot]Bushuven[at]glkn.de, an Ihre Krankenhaushygiene und Ihre medizinischen Ausbilder.

Mehr zur Händehygiene finden Sie zudem im Netz unter "WHO my 5 Moments" und der "Aktion Saubere Hände".

Die Umfrage ist beendet. Vielen Dank für die Teilnahme.

Das Fenster kann nun geschlossen werden.
