# Supplementary material for: Speaking up behavior and cognitive bias in hand hygiene: Competences of German-speaking medical students
Source: PLoS One. 2020 Sep 28;15(9):e0239444. doi: 10.1371/journal.pone.0239444 (PMC7521694; doi:10.1371/journal.pone.0239444)
Supplement: Attachment — Submitted filename: Response to the reviewers Letter PLoS One.docx [file pone.0239444.s003.docx]

Stefan Bushuven MD MSc DESA EDIC

Institut für Krankenhaushygiene und Infektionsprävention

Hausherrenstrasse 12 D-78315 Radolfzell, Germany

Phone: 0049 7731 89 2012 S.Bushuven@gmx.de

**To:**	Editorial Board of PloS One	August 15th, 2020

Dear Prof. G. Moore,

I am pleased to submit the revision of our research article entitled “***Speaking Up Behavior and Cognitive Bias in Hand Hygiene: Competences of German-speaking Medical Students***” for consideration for publication in PLOS.

With regard to the reviewers’ comments we made the following changes:

1. We changed the **style of the manuscript** according to the PLOS requirements
2. We added *the survey* (in German) as a pdf (supplemental material). The English translation of all 36 items can be found in the revised table 1.
3. We included the **data as an excel sheet** with anonymization of the participating universities as necessary to the study protocol
4. We changed the **title** according to the reviewers’ suggestions
5. We modified the **corresponding address** due to the main investigator’s work place changes
6. We made some **orthographic and stylistic corrections** as suggested
7. We defined **exclusion criteria** for “whole dropouts” not included in the Non-completer groups.
8. We **corrected the number of universities** participating from 11 to 12. This 12^th^ university had only 3 participants, but was included in the analysis.
9. We included a short **description of causes for overconfidence**
10. We included the two **tables** in the main manuscript. Figures remained in a separate file.

We hope to meet the requeirements for publication in PLOS One.

Please do not hesitate to contact us for further corrections on this manuscript.

Thank you for your consideration!

Sincerely, Stefan Bushuven, MD MSc
